# Atypical Change Detection in Sound Sequences: A Behavioral and Magnetoencephalography Study in Congenital Amusia

**DOI:** 10.1111/ejn.70529

**Published:** 2026-05-04

**Authors:** Yohana Lévêque, Camille Fakche, Lesly Fornoni, Françoise Lecaignard, Sébastien Daligault, Claude Delpuech, Julien Jung, Barbara Tillmann, Anne Caclin

**Affiliations:** ^1^ Université Claude Bernard Lyon 1, INSERM, CNRS, Centre de Recherche en Neurosciences de Lyon CRNL U1028 UMR 5292 Bron France; ^2^ CERMEP, MEG Department Bron France; ^3^ Functional Neurology and Epileptology Department Hospices Civils de Lyon Lyon France; ^4^ Université Bourgogne Europe, CNRS, LEAD UMR 5022 Dijon France

**Keywords:** loudness, MMN, pitch, prediction, sequencing, tone deafness

## Abstract

Pitch change detection and pitch memory are behaviorally impaired in congenital amusia. Yet rather preserved mismatch negativity (MMN) to unexpected pitch changes has previously been reported using EEG, suggesting a discrepancy between conscious and preattentive pitch perception in this population. Coupling MEG with EEG, our study re‐examined MMN in congenital amusia in light of two factors: stimulus onset asynchrony (SOA) and change size of the deviant. Individuals with and without congenital amusia passively listened to oddball sequences with either frequency (pitch) or intensity (loudness) deviants, using short (500 ms) or long (1500 ms) SOAs and small or large changes (0.25 or 2 semitones; −5 or −15 dB). In a subsequent active change detection task, participants with amusia had impaired detection of small frequency changes, while a smaller group difference was found for small intensity changes. Long SOAs increased amusics' behavioral response times more than those of controls for frequency and intensity deviants. Time courses of source data in MEG revealed decreased amplitude and increased latency of MMNs to frequency deviants in right temporal and right frontal cortices in amusia, across all the tested SOAs and change sizes. Some MMN abnormalities were found in amusic participants also for intensity deviants, across all the tested SOAs and change sizes. Thanks to the sensitivity of MEG, this study pinpoints that the right‐sided fronto‐temporal anomalies characterizing amusia are linked to modifications in the processing of sounds in sequences, most particularly for the pitch dimension, already at the preattentive level.

## Graphical abstract

When passively listening to oddball sequences during magnetoencephalography recordings, individuals with congenital amusia show abnormalities of mismatch negativity for frequency deviants in the right temporal and right frontal cortices, whatever the stimulus onset asynchrony and change size. More limited MMN abnormalities were observed for intensity deviants. During active detection of deviant sounds, behavioral measures reveal difficulties for small frequency (and intensity) changes in amusia. Increasing SOA has more impact on response time in participants with amusia than in controls.

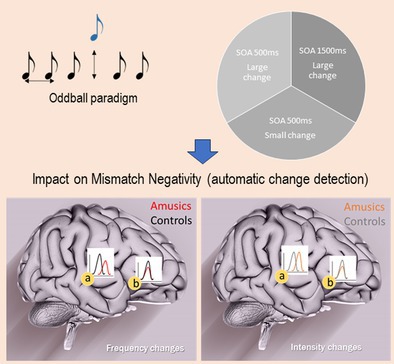

**Abbreviations**
EEG: electroencephalography
ERP/ERF: event‐related potential/field
fMRI: functional magnetic resonance imaging
IFG: inferior frontal gyrus
MBEA: Montreal Battery of Evaluation of Amusia
MEG: magnetoencephalography
MMN: mismatch negativity
PDT: pitch discrimination threshold
ROI: region of interest
SOA: stimulus onset asynchrony
STG: superior temporal gyrus

## 1 Introduction

Altered pitch processing and memory are hallmark features of congenital amusia. This lifelong neuro‐developmental disorder of music perception and production affects 1.5% of the population (Peretz and Vuvan 2017). Associated with a genetic component (Peretz et al. 2007), congenital amusia occurs without hearing loss, cognitive deficit, or brain injury, unlike acquired amusia following brain damage, and it is not due to a lack of environmental stimulation with music. In everyday life, amusic listeners have difficulties identifying when someone sings out of tune, including themselves, detecting wrong or out‐of‐key notes in a melody, memorizing melodies, even short ones, and recognizing familiar tunes without lyrics (for a recent review, see Tillmann et al. 2023).

Seminal studies of congenital amusia have highlighted that altered pitch processing characterizes this disorder (Ayotte et al. 2002; Hyde and Peretz 2004; Peretz et al. 2002) and suggested that an impaired discrimination of small pitch changes underlies the difficulty to process musical sounds. Critically, several studies revealed that amusic listeners had difficulties in short‐term memory tasks requiring the comparison of melodic sequences, even when the pitch changes between tones were higher than their individual pitch discrimination threshold (PDT), and even for amusics who had a PDT in the range of control participants' thresholds (Foxton et al. 2004; Tillmann et al. 2009; Albouy et al. 2013; review in Tillmann et al. 2016, 2023). Therefore, the hypothesis emerged that an impairment in pitch short‐term memory could be the core deficit in congenital amusia, beyond a pitch discrimination deficit.

In support of this hypothesis, behavioral studies demonstrated that amusic individuals were more impaired than controls when memory features were manipulated in short‐term memory paradigm with short sequences of tones. Congenital amusics' performance decreased more strongly than that of matched control participants when the retention delay between two tones increased, when there were interferent tones during the retention delay, and when memory load increased (Gosselin et al. 2009; Williamson et al. 2010; Williamson and Stewart 2010). The hypothesis of impaired pitch short‐term memory is also supported by results from anatomical and functional neuroimaging studies. In healthy participants, neuroimaging studies showed that encoding of pitch sequences in memory notably involves the bilateral superior temporal gyri (STG), including bilateral Heschl's gyri and planum polare, and the bilateral inferior frontal gyri (IFG) (Schulze et al. 2009; Griffiths 2001; Zatorre et al. 1994; Gaab et al. 2003). Using transcranial magnetic stimulation, Tse et al. (2018) also demonstrated the key role of fronto‐temporal network connectivity in pitch change detection. In congenital amusia, anatomical abnormalities were pinpointed in this network: modified gray and white matter proportion in the right STG (Hyde et al. 2007; Albouy et al. 2013) and the right IFG (Hyde et al. 2006; Albouy et al. 2013), reduced connectivity in the arcuate fasciculus, connecting dorsally the STG and the IFG (Loui et al. 2009; but see also Chen et al. 2015), altered depth of sulci in right IFG (Liao et al. 2022), with anatomical abnormalities increasing with the size of the musical deficit. Connectivity abnormalities in the right fronto‐temporal pathway were described in fMRI (e.g., Hyde et al. 2011) and magnetoencephalography (MEG) (Albouy et al. 2013; Albouy et al. 2015; Samiee et al. 2022) during encoding, retention, and retrieval of musical information. Functional anomalies were reported not only for active music perception and pitch short‐term memory tasks, but also for passive listening or the resting state brain in silence. Indeed, passive listening of a musical sequence was associated to reduced activation of the right IFG in amusic listeners compared with controls (Hyde et al. 2011) and underconnectivity within the fronto‐temporal network was described in the resting brain (Leveque et al. 2016; Jin et al. 2024).

Congenital amusia is thus characterized by anatomical and functional abnormalities in a fronto‐temporal pathway, which leads to behavioral difficulties at processing and memorizing pitch. However, while the topographic dimension of the cerebral abnormalities has been well described, the dynamic processes altered in congenital amusia remains debated. At which level of processing does the deficit arises? Are implicit and explicit musical processes impaired? Congenital amusics' abilities differ between implicit and explicit musical tasks: while their performance in familiar music recognition (e.g., Ayotte et al. 2002; Peretz et al. 2002; Tillmann et al. 2014) or explicit tonality judgments (Omigie et al. 2012; Tillmann et al. 2016) were impaired, it did not differ from that of controls in more indirect, implicit tasks (e.g., using priming tasks or feeling of familiarity judgments instead of recognition; see also Tillmann et al. 2012), even when using the same musical stimuli. These behavioral findings are in agreement with an EEG study revealing that although amusic individuals were not able to identify explicitly an out‐of‐tune note in a melody, there were still brain responses to musical incongruences, notably the N200 (Peretz et al. 2009; see also Zendel et al. 2015). Amusic individuals show a different evoked response to notes with high and low probability, suggesting they are sensitive to implicit rules of musical grammar (Omigie et al. 2013). These results collectively suggest that implicit processes could be more preserved than explicit ones in congenital amusia (see Tillmann et al. 2023 for a review).

Our study aimed to better understand to what extent pitch‐related implicit and explicit processes are impaired or preserved in congenital amusia. To this aim, we used an “oddball” paradigm, composed of auditory sequences with standard tones repeated a great number of times and unexpected tones with an occurrence frequency of 16%, called deviants. Seminal studies have shown that even passive listening of this “oddball” auditory paradigm elicits automatic event‐related potentials/fields in EEG/MEG (ERP/ERF), notably the mismatch negativity (MMN) (for a review, see Näätänen et al. 2007). The MMN is measured in the difference between the responses evoked by the deviant tone and the standard tone. The deviant tone violates a prediction from the central auditory system, based on the sensory memory trace of the previous sounds heard. Thus, the MMN has been interpreted as reflecting implicit sensory memory processes in auditory perception (for a review, see Näätänen et al. 2011). To allow for investigating the potential difference in implicit and explicit processing of the same material, we also used the same tone sequences in an active task where participants had to behaviorally identify deviant tones in the auditory stream.

Several previous studies used the oddball paradigm to investigate automatic detection of changes in sound sequences thanks to the MMN in congenital amusia. When out‐of‐tune deviants were placed into familiar melodies, MMN was absent in amusic participants in the study of Braun et al. (2008). However, the use of familiar melodies requires long‐term knowledge of music, which can be impaired in congenital amusia (see Ayotte et al. 2002; Graves et al. 2019 for behavioral data). Moreau et al. (2013) used a more classical implementation with repetitive standard tones where the deviant tone was played a quarter or two semitones from the standard tone. They reported no deficit in amusic participants, neither in MMN amplitude or latency. Converging evidence for relative MMN preservation in amusia has been gathered by studies using different auditory contexts and materials (Pralus et al. 2020 for neutral and emotional prosody; Nan et al. 2016 and Zhang and Shao 2018 for lexical tones; Graves et al. 2023 for dissonant pitches; Quiroga‐Martinez et al. 2021, 2022 for pitch sequences). The MMN could be reduced in amusia in more complex sequences than simple oddball paradigms (Quiroga‐Martinez et al. 2021), but overall these studies support the hypothesis that implicit processing of pitch is not impaired or less impaired than explicit processing in amusia. Congruently, amusics' pitch processing impairment is reflected in later ERPs associated with conscious detection of deviance, such as reduced or delayed P3 (Pralus et al. 2020; Graves et al. 2023) or P600 (Peretz et al. 2009). Up to now however, no study has used MEG to shed light on the process of automatic pitch change tracking in amusia, yet MEG enables the study of MMN at the source level, with a high temporal resolution and a high SNR (e.g., Lecaignard et al. 2021). Two previous MEG studies (Albouy et al. 2013, 2015; Samiee et al. 2022) explored the phenomenon of congenital amusia with active sequence processing. During active encoding of musical sequences in memory, MEG recordings revealed a reduced amplitude and delayed latency of the N100m, in the IFG and STG, suggesting an early deficit during the auditory processing stream in congenital amusia (Albouy et al. 2013). Phase‐amplitude coupling anomalies were also described between the auditory cortex and the motor and frontal inferior cortex during active pitch change detection (Samiee et al. 2022). To our knowledge, the present study investigates for the first time passive listening in congenital amusia with MEG.

The integrity of pitch MMN in amusia could be conditioned by the time interval between two stimuli (stimulus onset asynchrony [SOA]). Albouy et al. (2016) brought to light that amusic performance was impaired in a pitch discrimination task and a pitch sequence short‐term memory task when the SOA was set to 100 ms, while their performance in both tasks was in the range of those of controls for a longer SOA of 350 ms or more (Albouy et al. 2016). Furthermore, the sound memory trace could be more fragile and time‐sensitive in amusic individuals than in controls, with a deleterious effect of a long SOA to discriminate pitches. For instance, amusics' performance was highly altered in a pitch discrimination task when the SOA increased, from 1 to 15 s (Williamson et al. 2010). These results suggest that there is a preferential SOA in congenital amusia to process sounds, somewhere between 350 and 1 s. Yet in their MMN paradigm, Moreau et al. (2013) used a 500‐ms SOA, which thus may not allow observing an alteration of the memory trace among amusics. Additionally, at the behavioral level, amusic individuals are mostly impaired in pitch discrimination tasks when the pitch change is small (Ayotte et al. 2002; Hyde and Peretz 2004).

The present study manipulated size of change and SOA as possible modulators of MMN differences between amusic participants and matched controls. In typical listeners, both pitch size change and SOA influence MMN amplitude and latency (e.g., He et al. 2009): increasing SOA increases MMN latency and decreasing pitch size change decreases MMN amplitude. We hypothesized that both factors would be more deleterious to amusics' MMN than to controls' one. Specifically, to study the pitch discrimination deficit among amusics, we used (1) a reference condition with a large deviance (two semitones) and a 500‐ms SOA, which seems favorable to limit the impact of pitch‐short term memory difficulties in congenital amusia and where we expected a MMN; (2) a small deviance of a quarter of semitone, associated to a 500‐ms SOA, to emphasize fine‐grained discrimination difficulties (Moreau et al. 2013; Albouy et al. 2016); (3) a large deviance of two semitones associated to a long SOA of 1500 ms with the aim to highlight the time‐dependent pitch short‐term memory deficit in amusia. We expected an alteration of the MMN for the small deviance conditions and the long SOA, that is, a pitch processing impairment at an implicit, automatic level among amusics. With an active part of the protocol using the same conditions and sequences (but in shorter blocks), we aimed to confirm the explicit deficit in pitch discrimination and short‐term memory in congenital amusia, and compare automatic detection of deviants as measured with the MMN to active detection of the same deviants. Additionally, in the passive as well as the active task, we used deviants in intensity (in addition to those in frequency) to confirm the abilities of amusics to process the information related to sound amplitude (Cousineau et al. 2015) and exclude general impairments in sound change detection.

To investigate automatic change detection in amusia as described above, we used paired MEG‐EEG recordings. MEG can detect differences between amusic and control participants in the early, obligatory evoked response, as described for the N1 component during an active pitch short‐term memory task (Albouy et al. 2013, 2015) and in network dynamics during active pitch change detection (Samiee et al. 2022). We performed source reconstruction to investigate if and where there are network activation differences between amusics and controls in the MMN time window. For a greater sobriety in results presentation, this article focuses on the MEG data, and EEG results are presented in Supporting Information for comparability with previous studies.

## 2 Methods

### 2.1 Participants and Behavioral Pretests

Thirteen congenital amusic adults and 12 control participants were enrolled in the study. Three participants were excluded from the analyses: one control participant whose behavioral performance was two standard deviations below the mean of their group, one amusic participant with unusable MEG recordings because of metallic dental prostheses that generated ferromagnetic artifacts, and one amusic participant who was unable to finish the protocol. The final group was composed of 11 amusic and 11 control participants, matched in age, sex, handedness, education, and musical education (see Table 1). All participants reported no history of neurological or psychiatric disease. They also had normal hearing; that is, they all had no more than 20‐dB hearing loss in any ear on average at 500, 1000, 2000, and 4000 Hz, as tested with a portable Colson K15 audiometer with TDH39 headphones (Table 1). Participants gave their written informed consent and were paid for their participation. Ethical approval was obtained from the national ethics committee on Human Research (CPP SUD‐EST IV, #2012‐A01209634) according to French regulation.

**TABLE 1:** Participants' demographic data. Education was counted from the first year of elementary school, which begin at the age of six in France. Musical education corresponded to years spent at receiving musical instrument lessons. MBEA score was the average score over the six subtests of the MBEA. Pitch score corresponded to the average scores obtained at the three MBEA subtests that investigate pitch perception (Scale, Contour, Interval, as, e.g., in Williamson et al. 2010). Average auditory thresholds were calculated from those at frequencies 500, 1000, 2000, and 4000 Hz. We considered that an auditory loss took place when the average auditory threshold of a subject differed from the population norm, that is, was above 20 dB. Mean comparisons between the two groups of the demographic variables were done with two‐tailed Student *t* tests in heteroscedasticity conditions, and ratio comparison on laterality was done thanks to a Fischer exact test. SD: standard deviation, Min.: minimum value, Max.: maximum value.

	Amusics				Controls				Group difference *p*
Number of participants	11				11				
Ratio male/female	4/7				4/7				
Ratio right/left hander	10/1				9/2				1
	Mean	SD	Min.	Max.	Mean	SD	Min.	Max.	
Age (years)	40.38	14.75	21	62	37.97	11.87	24	57	0.68
Education (years)	13.91	2.7	10	20	14.73	2.33	12	20	0.46
Musical education (years)	0.73	1.42	0	4	0.23	0.52	0	1.5	0.29
MBEA mean score (maximum score = 30)	21.11	0.99	19	23	27.39	1.54	24.5	30	< 0.0001
MBEA Pitch score (maximum score = 30)	20.18	1.89	18	24.67	26.7	2.22	22	30	< 0.0001
PDT (semitones)	0.84	0.69	0.2	2.41	0.25	0.17	0.09	0.71	0.02
Right ear auditory threshold (dB)	7.61	4.63	1.25	18.75	7.95	4.48	−2.50	13.75	0.86
Left ear auditory threshold (dB)	9.20	4.38	2.50	13.75	6.93	6.26	−5.00	11.25	0.34

To be considered as congenital amusic, participants had to obtain at the Montreal Battery of Evaluation of Amusia (MBEA, Peretz et al. 2003) an average score that was two standard deviations below the mean of the normal population (i.e., a cut‐off score of 23, maximum score = 30). In the MBEA, six subtests address various components of music perception and memory, notably the pitch dimension (detection of an out‐of‐key note, a contour change, or an interval change), the time dimension (rhythm and meter perception), and incidental memory (i.e., recognizing melodies used in preceding subtests). All participants were tested with the MBEA, and all participants of the control group performed above the cut‐off scores (Table 1).

In addition to the MBEA, a PDT was measured for each participant with a two‐alternative forced‐choice task based on an adaptive tracking, two‐down/one‐up staircase procedure (Tillmann et al. 2009). On average, thresholds of amusic participants were significantly higher (worse) than thresholds of control participants, even though there was an overlap between groups (Table 1).

### 2.2 Stimuli and Sequences

**FIGURE 1:**
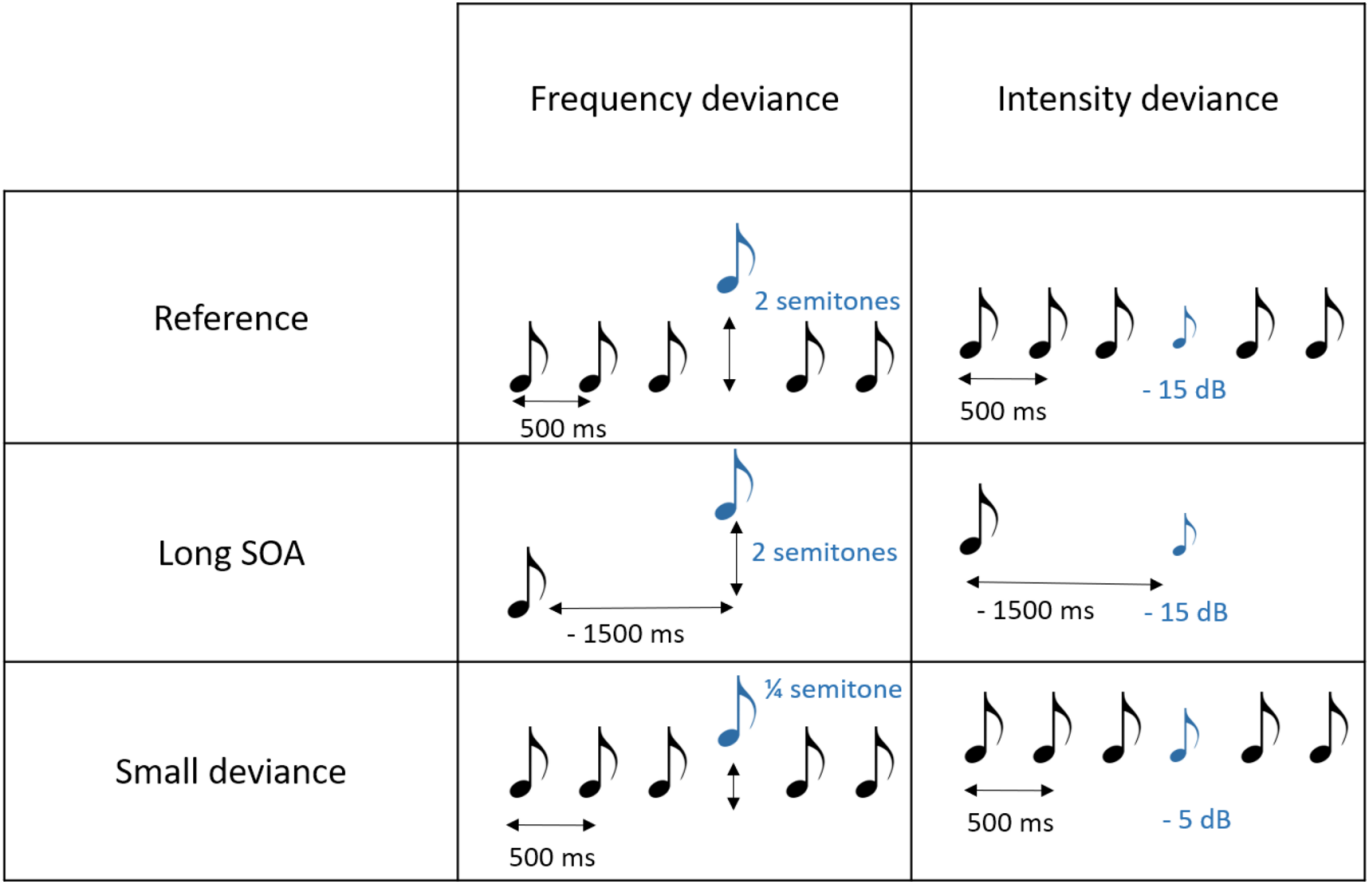
Six auditory sequences. There were three conditions with frequency deviance, and three with intensity deviance. For the Reference conditions, a 500‐ms SOA and a large deviance of two semitones (frequency) or −15 dB (intensity) are used; for the Long SOA conditions, a 1500‐ms SOA and a large deviance are used; and for the small deviance conditions, a 500‐ms SOA and a Small deviance of a quarter of semitone (frequency) or −5 dB (intensity) are used.

Synthetic harmonic sounds, composed of the fundamental frequency F0 and its first three harmonics with decreasing amplitude, were used. Sound duration was 100 ms, including 5‐ms rise‐time and 5‐ms fall‐time. The sounds were used in six auditory sequences. In each sequence, the standard tone was the same, played at a frequency level of C6 (1047 Hz), and there was a specific deviance characterized by its acoustics feature, that is, a large or a small change in frequency or in intensity, and a fixed SOA. For large deviances, for each feature (frequency and intensity), one sequence had a SOA of 500 ms, and another sequence a SOA of 1500 ms. For small deviances, the SOA was fixed to 500 ms (Figure 1).

Deviant tones in frequency were played a quarter of a semitone higher than the standard tone for the small deviance (1062 Hz), and two semitones higher for the large deviance (1175 Hz, frequency level of D6). We chose a small deviance in frequency of a quarter of a semitone because amusics' performance to detect this change was close to chance and clearly inferior to controls' performance (see also Hyde and Peretz 2004). Deviant tones in intensity were played 5 dB below the standard tone for the small deviance, and 15 dB below for the large deviance. These values were fixed after pilot testing with the aim to get equivalent levels of difficulty in discriminating frequency and intensity deviants in the reference condition (large deviance, short SOA). A difference of −5 to −15 dB was also used in the work of Tervaniemi et al. (1999). An attenuation was chosen for intensity deviance because an augmentation tends to increase the N1‐wave amplitude, which overlaps with the MMN and would lead to overestimate it. In each auditory sequence, the probability of hearing a deviant was 16.7% and deviant tones occurred after three to seven standards.

### 2.3 Procedure

Participants performed the entire passive and active listening protocol during simultaneous MEG and EEG recordings. They were seated in a sound‐attenuated, magnetically shielded recording room, and listened to the sounds presented binaurally through air‐conducting tubes with foam ear tips. Participants were asked to avoid head's movements and eye blinking. Before the beginning of the MEG/EEG recordings, each participant's detection threshold for the standard tone was determined, and the intensity level was adjusted so that the sounds were presented 45 dB above their threshold. Participants began with passive listening conditions, during which they were watching a self‐selected muted movie with subtitles. They were told to ignore the sounds so that their attention was focused on the visual stimulation. Then, they performed the active task, where they were instructed to press a button when they detected a deviant tone. Beginning with the passive conditions allowed keeping subjects naïve and avoid that they searched for deviant tones. The order of presentation of the six types of auditory sequences was counterbalanced across participants according to a Latin square controlling for first‐order carry‐over effects. *Presentation* software (Neurobehavioral systems) was used to present stimuli and record participants' button presses.

Passive and active blocks differed in terms of the instruction given but also in terms of the number of trials. For the passive recordings, auditory sequences with 500‐ms SOA contained 840 stimuli including 140 deviant tones, and those with 1500‐ms SOA, 280 stimuli including 47 deviant tones. Each sequence lasted ~7 min. One sequence was presented for each condition, except for sequences with 1500 ‐ms SOA where three sequences were presented in a row to obtain the same number of deviant tones as in the other conditions for the analyses (i.e., 140 deviants per condition), leading to ~70 min of recordings in passive sequences. There were short breaks between sequences. In contrast, auditory sequences of the active task were composed of 180 stimuli including 30 deviants for each condition, for a total duration of 17 min.

### 2.4 Behavioral Data: Preprocessing and Statistical Analysis

To study participants' behavioral abilities, we extracted the number of hits, false alarms, and reaction times with custom MATLAB scripts (The MathWorks Inc.). We considered that hits were comprised in a time window of [+150; +1500] ms post‐stimulus, answers given earlier (i.e., by anticipation) or later being considered as false alarms. ANOVAs were run with the *Statistica* software (StatSoft). *p* values were adjusted with a Greenhouse–Geisser correction when needed. Least significant difference (LSD) Fisher post hoc tests were run to refine the interpretation of significant main effect and interactions. For false alarms, Mann–Whitney *U* tests were used to compare the Groups (Amusic/Control) in each of the six conditions, as data were skewed, with, in each condition, at least 10 participants having no false alarms.

### 2.5 MEG Recordings

Recordings were carried out using a 275‐channel whole‐head MEG system (CTF‐275 by VSM Medtech Inc.) and a 56‐electrode EEG system incorporated with the MEG system (see Supporting Information for methods and results concerning EEG). Horizontal and vertical electrooculograms (EOG) and electrocardiogram (ECG) recordings were acquired with bipolar montages. Electrophysiological recordings were made continuously with a sampling rate of 600 Hz and a 0.016‐ to 150‐Hz bandwidth. A MEG gradient correction (first order) was applied to the data. Head position was determined continuously (150‐Hz sampling rate) with coils fixated at three fiducials points: the right and left preauricular points, and the nasion. Electrodes and fiducials positions were localized on the participant's head prior to the recordings using a digitization stylus (Fastrak, Polhemus). Furthermore, T1‐weighted magnetic resonance imaging images (MRIs) of the head were obtained for each participant. High MRI contrast markers were placed at fiducial positions to facilitate their pointing on MRIs and hence anatomical and functional data coregistration.

### 2.6 MEG Data Preprocessing and ERF Computation

For the passive listening data, MEG data were first preprocessed using CTF tools (CTF MEG International Services LP), Matlab scripts (The MathWorks Inc.), and the ELAN software package developed at the Lyon Neuroscience Research Center (Aguera et al. 2011). For the active task data, there was no analysis of the MEG/EEG data: Only behavioral analyses were performed.

A median head position was determined for each participant, across all conditions, thanks to the recordings of fiducials points' positions. With respect to the head position established, we excluded the epochs contaminated by head's movements higher than 1.5 cm from the median position. Then, we identified SQUID jumps, that is, for MEG data, when the signal exceeds a 10,000‐fT threshold on any 400‐sample‐long time window. SQUID jumps were corrected and the epochs containing SQUID jumps were excluded from the analyses afterward.

One amusic participant and one control participant presented cardio‐respiratory artifacts on their MEG recordings. Thus, for these two participants, we applied a signal correction model with a signal space projection method provided by CTF and described in Albouy et al. (2013). This method allows uncovering a signal‐space vector that describes the global topography of the artifact in sensor space. A projection of the MEG data onto the space orthogonal to this vector was then applied to remove the artifact.

For all participants, bandstop filters centered at 50, 100, and 150 Hz were applied to the continuous data to remove power‐line artifacts. Continuous data were then filtered between 2 and 45 Hz (third‐order Butterworth filters). Epochs were extracted from −100 to 400 ms around each stimulus onset. Epochs with signal variations at any sensor over 2000 fT (MEG sensors), 100 μV (EEG sensors), or 50 μV (EOG sensors) were discarded, and noisy EEG electrodes were interpolated using spherical splines. For each participant, each condition, and each sensor, ERFs were obtained by averaging all artifact‐free epochs separately for standard and deviant tones (discarding the first three standards of each block as well as standards immediately following deviants). On average across conditions, the number of epochs averaged was: 121 ± 15 (mean ± SD across participants) for deviants in the amusic group; 481 ± 64 for standards in the amusic group; 118 ± 15 for deviants in the control group; 470 ± 64 for standards in the control group. Baseline correction was done by subtracting the mean of the signal in the −100‐ to 0‐ms period of stimulus onset. Difference ERFs were then computed by subtracting the standard ERF to the corresponding deviant ERF for each participant and condition.

### 2.7 MEG: Source Reconstruction

The individual T1‐weighted MRIs were segmented using the FreeSurfer software package (Fischl 2012; http://surfer.nmr.mgh.harvard.edu). After visual inspection, segmented files were imported in the Brainstorm toolbox (Tadel et al. 2011; http://neuroimage.usc.edu/brainstorm) for source analyses, along with difference ERFs and positions of MEG sensors. A noise covariance matrix was computed on empty‐room recordings performed on the day of the experiment, preprocessed with the same pipeline as for the task. After coregistration between the individual anatomy and MEG sensors, the forward model was computed with OpenMEEG software (Gramfort et al. 2010; https://openmeeg.github.io/). A weighted minimum‐norm estimation was conducted in the source space coming from the white matter–gray matter boundary segmented by FreeSurfer. Specifically, cortical currents were estimated using a distributed model consisting of 15,002 current dipoles from the time series of the 275 gradiometer signals using a linear inverse estimator (weighted minimum‐norm current estimate, signal‐to‐noise ratio of 3, whitening PCA, depth weighting of 0.5). Source orientations were constrained to be orthogonal to the gray–white matter boundary of the individual MRIs. Source maps were then projected on a standard brain (ICBM152) and spatially smoothed (FWHM = 3 mm). This was followed by the group averaging and statistics.

### 2.8 Statistical Analysis of Source‐Level Data

**FIGURE 2:**
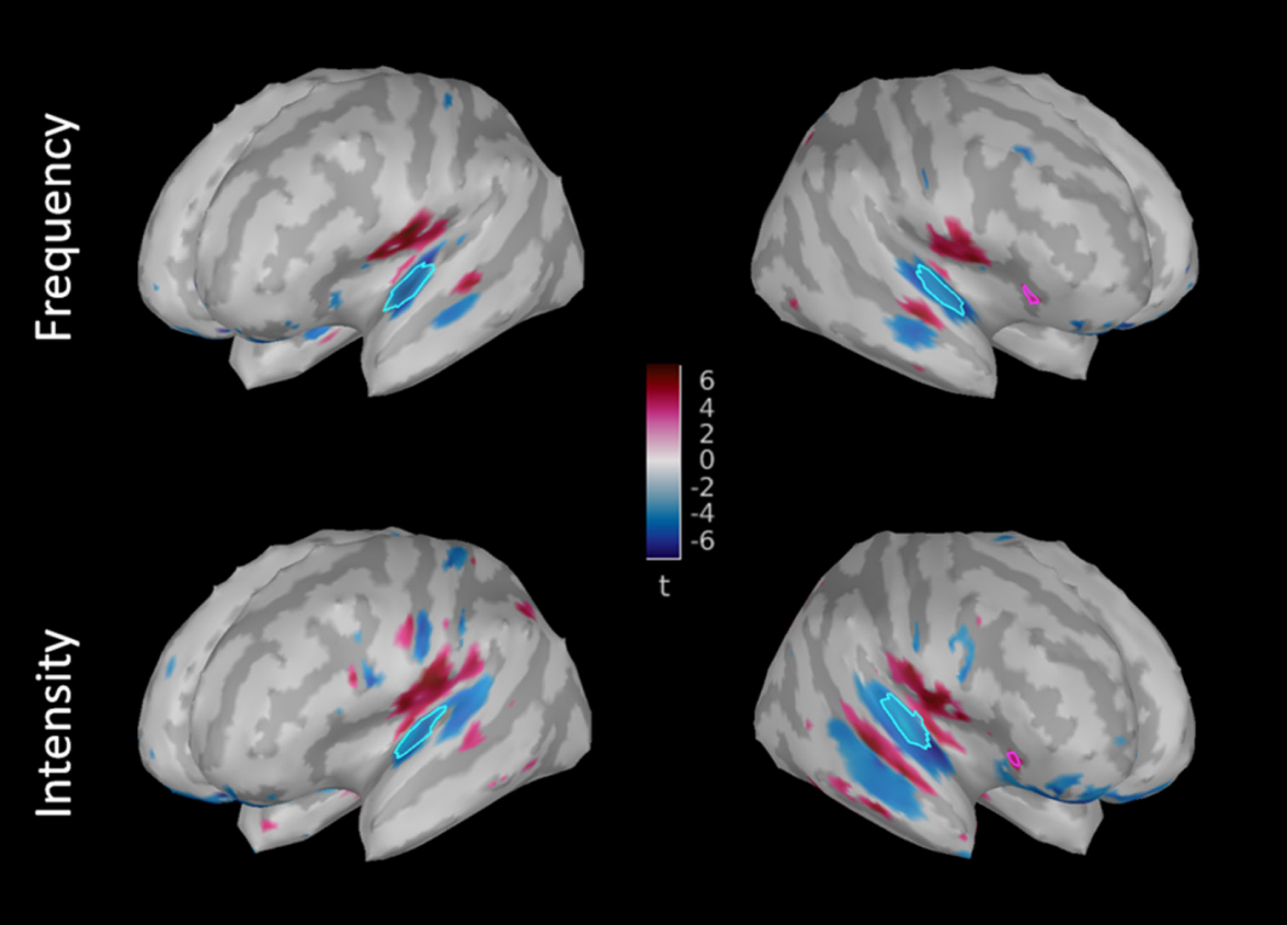
MMN sources on a 3D brain model. *t* values of emergence tests across the 22 participants (FDR‐corrected in space and time) are depicted on left and right hemisphere views. Temporal Regions of Interests (ROIs) are circled in light blue, and frontal ROIs are circled in pink. Sources were localized at the MMN peak (in the Reference Frequency condition, at latency 130 ms, top panels, and in the Reference Intensity condition, at latency 180 ms, bottom panels).

ERF analyses were performed following these steps:

1 Emergence of ERFs was tested at the whole brain level using t tests comparing signal amplitude to zero for each time sample at each vertex across all participants for each condition;
2 Regions of interest (ROIs) were drawn in the Reference conditions for Frequency and for Intensity at MMN peak latency: 130 ms for the Frequency and 180 ms for the Intensity Reference conditions (see Figure 2). The Frequency Reference condition ROI was used for all Frequency conditions and the Intensity Reference condition ROI was used for all Intensity conditions in subsequent analyses. ROIs were grown with the semiautomatic procedure implemented in Brainstorm. Temporal ROIs were grown in the superior temporal plane in each hemisphere, starting from a vertex with a significant MMN in the vicinity of Heschl's gyrus, and the right frontal ROI was grown from a vertex exhibiting a significant MMN in the inferior frontal gyrus, in close spatial correspondence to the frontal ROI in Lecaignard et al. (2021).

3 Temporal windows of interest where the MMN emerged were determined across all participants for each condition in the right temporal ROI. The right temporal region was chosen because it had the longest window of emergence (encompassing the others). In the Frequency Reference condition, the window was 85–178 ms, in the Frequency Long SOA condition: 105–177 ms, in the Frequency Small deviance condition: 151–238 ms; in the Intensity Reference condition: 138–213 ms; in the Intensity Long SOA condition: 150–221 ms; and in the Intensity Small deviance condition: 168–243 ms.
4 The mean amplitude and peak latency of time courses in this temporal window for each condition were extracted for each participant in each ROI.

Statistical analyses on MMN mean amplitude and peak latency (minimum or maximum depending on polarity of the response in a given ROI) were done with a 2 × 3 × 2 ANOVA with between‐participants factor Group, and within‐participant factors Condition (Reference/Long SOA/Small deviance) and Dimension (Frequency/Intensity), using the software Statistica on each ROI. *p* values were adjusted by a Greenhouse–Geisser correction when needed (in such cases, we report *ε* and corrected *p* values).

## 3 Results

### 3.1 Behavior

For the numbers of hits and response times, 2 × 2 × 3 or 2 × 2 × 2 ANOVAs were computed with Group (Amusic/Control) as a between‐participants factor, and Dimension (Frequency/Intensity) and either Condition (Reference/Long SOA/Small deviance; for hits) or SOA (Reference/Long SOA; for RTs) as within‐participant factors.

#### 3.1.1 Performance (Hits and False Alarms)

**FIGURE 3:**
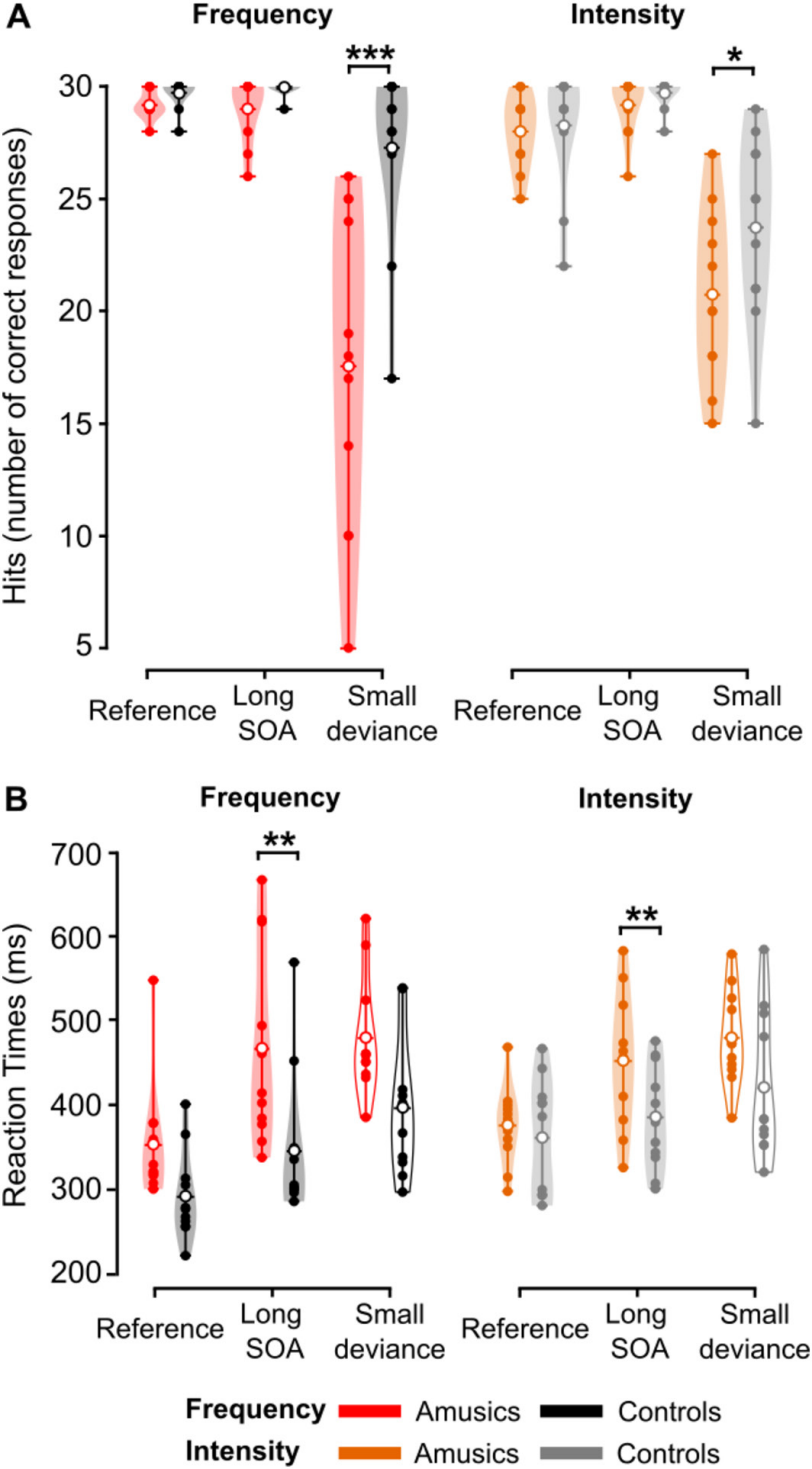
Behavior. (A) Number of hits and (B) reaction times (RTs) for frequency (left) and intensity (right) conditions in each group. The maximum number of hits was 30. RTs are for correct responses (Hits) only. RTs in the small deviance conditions are reported for completeness but were not analyzed due to the small number of Hits in some participants in these conditions (empty violin plots). ***: *p* < 0.001; **: *p* < 0.01; *: *p* < 0.05 (between‐group comparisons). Amusics, red and orange violin plots; controls, black and gray violin plots. Filled dots represent individual data, empty dots the mean for each group.

The ANOVA on number of Hits (Figure 3A) revealed a significant effect of Group (*F*(1, 20) = 14.15, partial *η* ^2^ = 0.414, *p* < 0.001), Condition (*F*(2, 40) = 62.86, partial *η* ^2^ = 0.759, *ε* = 0.590, *p* < 0.001), and significant interactions between Group and Dimension (*F*(1, 20) = 5.33, partial *η* ^2^ = 0.210, *p* = 0.03), Group and Condition (*F*(2, 40) = 11.34, partial *η* ^2^ = 0.362, *ε* = 0.590, *p* = 0.002), and a triple interaction between Group, Dimension, and Condition (*F*(2, 40) = 6.26, partial *η* ^2^ = 0.239, *ε* = 0.658, *p* = 0.01). The effect of Dimension and the interaction between Dimension and Condition were not significant (all *p*s > 0.35).

Post hoc analyses on the triple interaction revealed that amusics' and controls' hit rates were not significantly different for Reference and Long SOA conditions, for deviances in Intensity or in Frequency (all *p*s > 0.48). However, amusics' hit rates were significantly lower than those of controls for Small deviances in Frequency (*p* < 0.001) and in Intensity (*p* = 0.02). Furthermore, this analysis showed that amusics' performance was higher when detecting small deviances in intensity compared with deviances in frequency (*p* = 0.004), while it was the reverse for controls, with better performance for small deviances in frequency compared with deviances in intensity (*p* = 0.002).

False alarms (i.e., the participant pressing the button to detect a deviant while there is no deviant) were rare (< 2 per condition on average for each group). There was no significant difference between the amusic and control group for the false alarms in any of the six conditions (*p*s > 0.25, Mann–Whitney *U* tests).

#### 3.1.2 Reaction Times

We did not analyze RTs in the Small deviance condition as they could not be properly estimated due to a very low number of hits in some participants (< 10 in three amusic participants). Indeed, these participants made a lot of omissions (i.e., they did not press the button despite the presence of a deviant).

ANOVA was run with a Group factor, a SOA factor (Reference versus Long SOA) and a Dimension factor. The ANOVA on RTs (Figure 3B) yielded significant effects of factors Group (*F*(1, 20) = 7.13, partial *η* ^2^ = 0.263, *p* = 0.015), SOA (*F*(1, 20) = 28.60, partial *η* ^2^ = 0.588, *p* < 0.001), and of the interactions between SOA and Dimension (*F*(1, 20) = 5.77, partial *η* ^2^ = 0.224, *p* = 0.03), and Group and SOA (*F*(1.20) = 4.96, partial *η* ^2^ = 0.199, *p* = 0.04). The interaction between SOA and dimension revealed that participants were significantly slower to identify a large deviance in intensity compared with a large deviance in frequency when the SOA was set to 500 ms (*p* < 0.001), but not for the SOA of 1500 ms (*p* = 0.21). The interaction Group * SOA reflected the fact that amusics were significantly slower than controls for the Long SOA condition (*p* = 0.002), while it was not the case for the shorter SOA of 500 ms (*p* = 0.18).

The main effect of Dimension and the interaction between Group and Dimension were of large effect size but did not reach statistical significance (respectively *F*(1, 20) = 3.8, partial *η* ^2^ = 0.159, *p* = 0.07 and *F*(1, 20) = 2.75, partial *η* ^2^ = 0.121, *p* = 0.11). The triple interaction was not significant (*p* = 0.78).

### 3.2 Amplitude and Latency of the MMN in the Source Space

**FIGURE 4:**
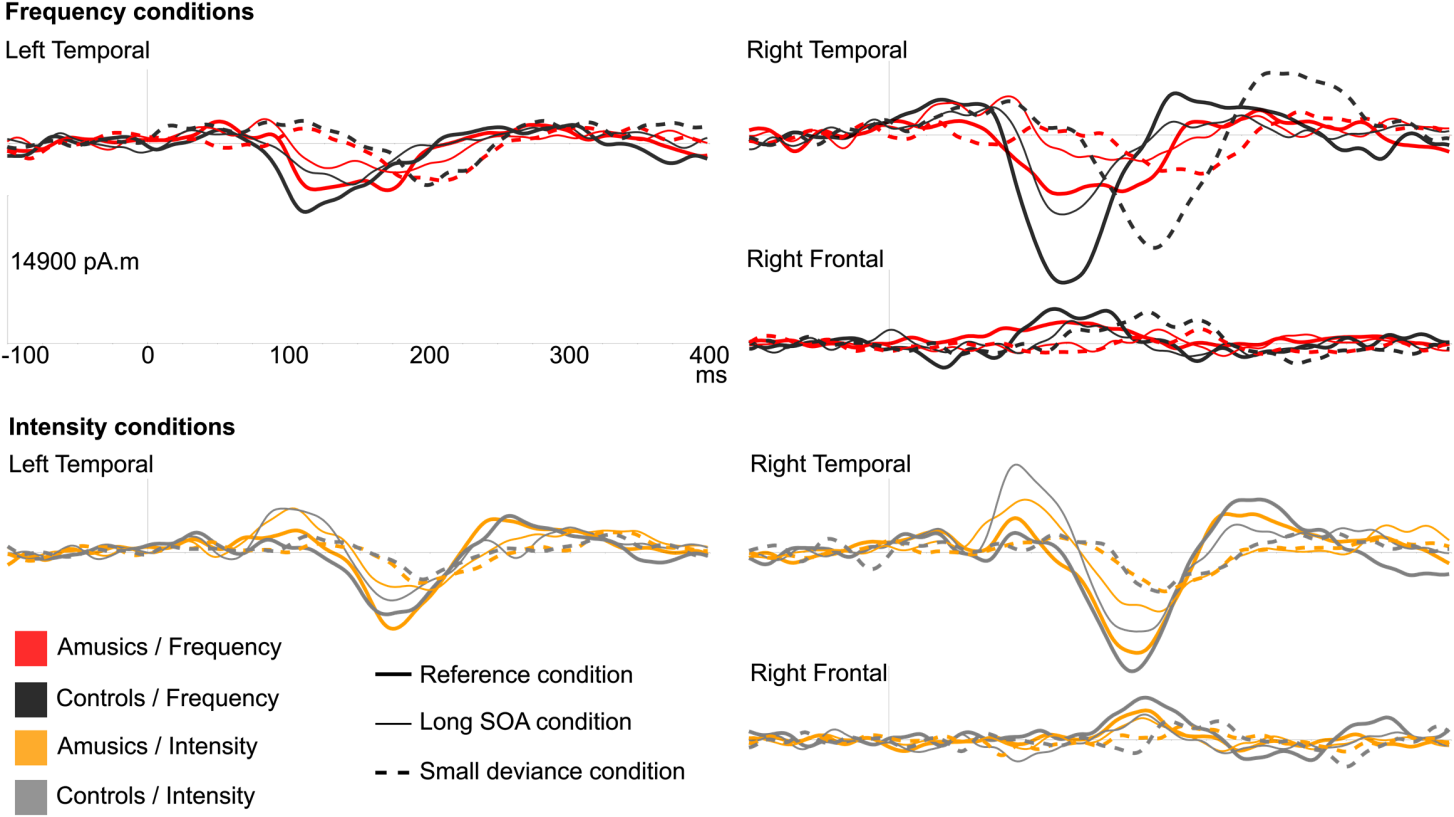
Grand average of time courses of source data for each ROI and condition, for each group. Event‐related responses (difference waves) are illustrated in the time window [−100 to 400 ms] around sound onset. Color code as in Figure 3 represents group and dimension of change. Frequency conditions are illustrated in the top panels, intensity conditions in the bottom panels. Line characteristics represent conditions (bold for Reference, plain for Long SOA, and bold‐dotted for Small change).

We extracted mean MMN amplitude (in the time‐window of interest) and MMN peak latency from the time courses of source‐level data, for each of the six experimental conditions and each participant, in the three ROIs for each dimension (right and left temporal, right frontal; see Figure 2). Minimum latencies were used for temporal regions and maximum latencies for frontal regions, according to the polarity of the event‐related responses (Figure 4). One amusic and one control participant had missing MEG data for one condition; we thus removed data from these two participants for the MEG data statistical analyses.

#### 3.2.1 Right Temporal ROI (Figure 5)

**FIGURE 5:**
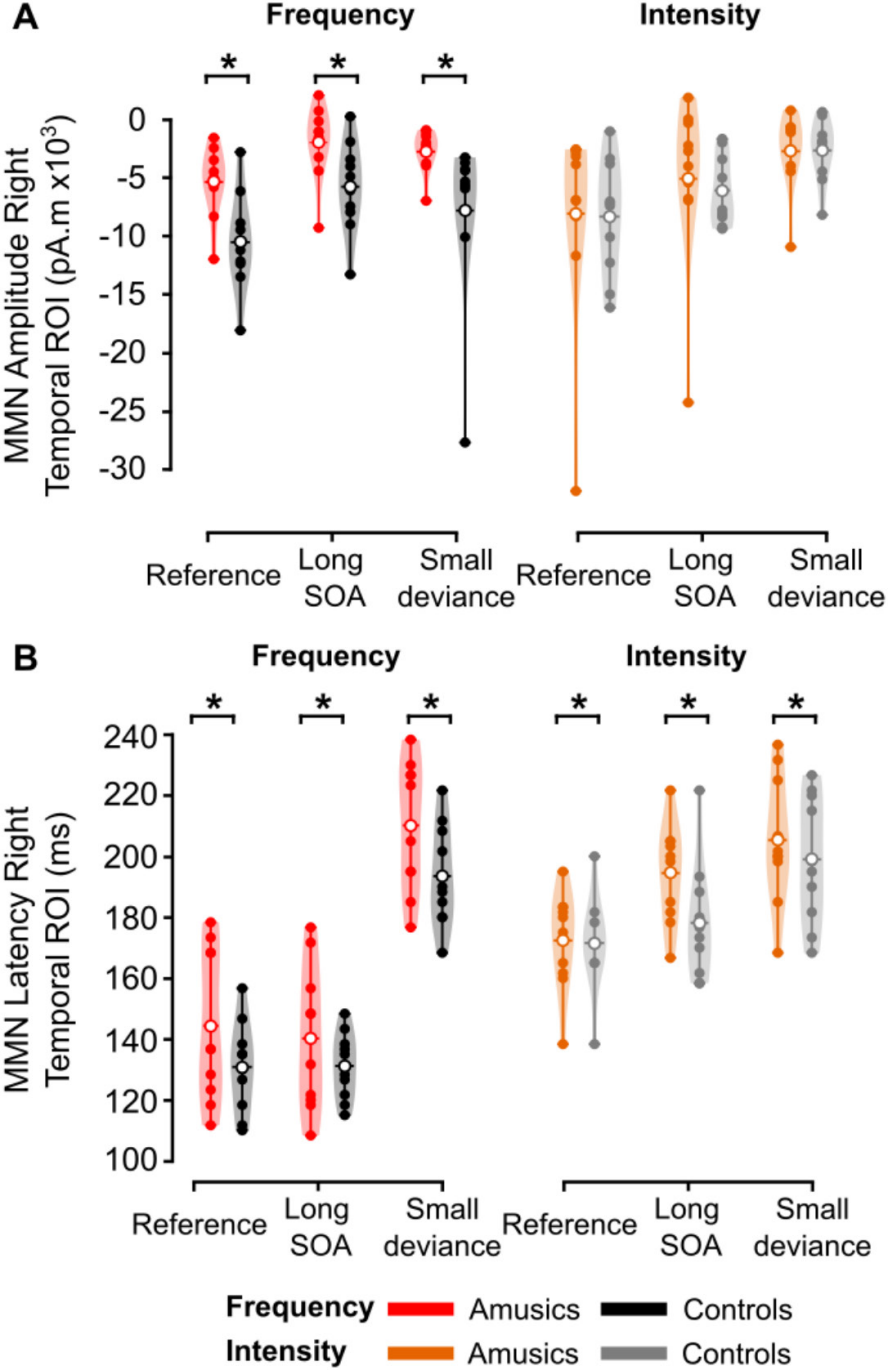
MMNs in the right temporal ROI. (A) Mean source amplitude (in pA·m) and (B) mean peak latency (in ms) in each group. Color code as in Figure 3 represents group and dimension of change: amusics, red and orange violin plots; controls, black and gray violin plots. Filled dots represent individual data, empty dots the mean for each group.

In the ANOVA on MMN amplitude, we found a significant effect of Condition (*F*(2, 36) = 26.29, partial *η* ^2^ = 0.594, *ε* = 0.781, *p* < 0.001): MMN amplitude at the source level was reduced for Long SOA (*p* < 0.001) and Small deviance (*p* < 0.001) conditions compared with the Reference condition, with no significant difference between the Long SOA and Small deviance conditions (*p* = 0.22). There was a significant interaction between Group and Dimension (*F*(1, 18) = 4.96, partial *η* ^2^ = 0.216, *p* = 0.039): amusic participants had a significantly lower MMN amplitude than controls for the Frequency deviances (*p* = 0.026), while MMN amplitudes were not different between groups for the Intensity deviances (*p* = 0.842). A significant interaction between Condition and Dimension (*F*(2, 36) = 7.19, partial *η* ^2^ = 0.286, *ε* = 0.749, *p* = 0.002) reflected the fact that MMN amplitudes were larger for Intensity deviants compared with Frequency deviants in the Long SOA condition (*p* = 0.043) and smaller for Intensity deviants compared with Frequency deviants in the Small deviance condition (*p* = 0.003), without a significant difference between dimensions in the Reference condition (*p* = 0.75). Other effects and interactions were not significant (*p*s > 0.163).

In the ANOVA on MMN latency, we found a significant effect of Group (*F*(1, 36) = 4.54, partial *η* ^2^ = 0.202, *p* = 0.047), with increased MMN latency in amusic participants compared with control participants. The effect of Condition was significant (*F*(2, 36) = 86.97, partial *η* ^2^ = 0.829, *ε* = 0.899, *p* < 0.001) with the Small deviance condition having significantly increased MMN latency compared with the Reference and Long SOA conditions (both *p* < 0.001), without a significant difference between the Reference and Long SOA conditions (*p* = 0.12). The effect of Dimension was also significant (*F*(1, 18) = 74.17, partial *η* ^2^ = 0.805, *p* < 0.001) and interacted with Condition (*F*(2, 36) = 26.55, partial *η* ^2^ = 0.596, *ε* = 0.956, *p* < 0.001). Intensity‐MMN had increased latency compared with Frequency‐MMN in the Reference condition (*p* < 0.001) and in the Long SOA condition (*p* < 0.001), but not in the Small Deviance condition (*p* = 0.95). Other effects and interactions were not significant (*p*s > 0.31).

#### 3.2.2 Left Temporal ROI (Figure S1)

On MMN amplitude and latencies, there was no significant Group effect and the Group factor did not interact with other factors (all *p*s > 0.15). While not reaching statistical significance, the interaction between Condition × Group for MMN latencies had a large effect size (*F*(2, 36) = 3.24, partial *η* ^2^ = 0.152, *ε* = 0.859, *p* = 0.06), reflecting an increased MMN latency in the Long SOA condition in amusics compared with controls. For detailed statistics on this ROI, see Supporting Information.

#### 3.2.3 Right Frontal ROI (Figure 6)

**FIGURE 6:**
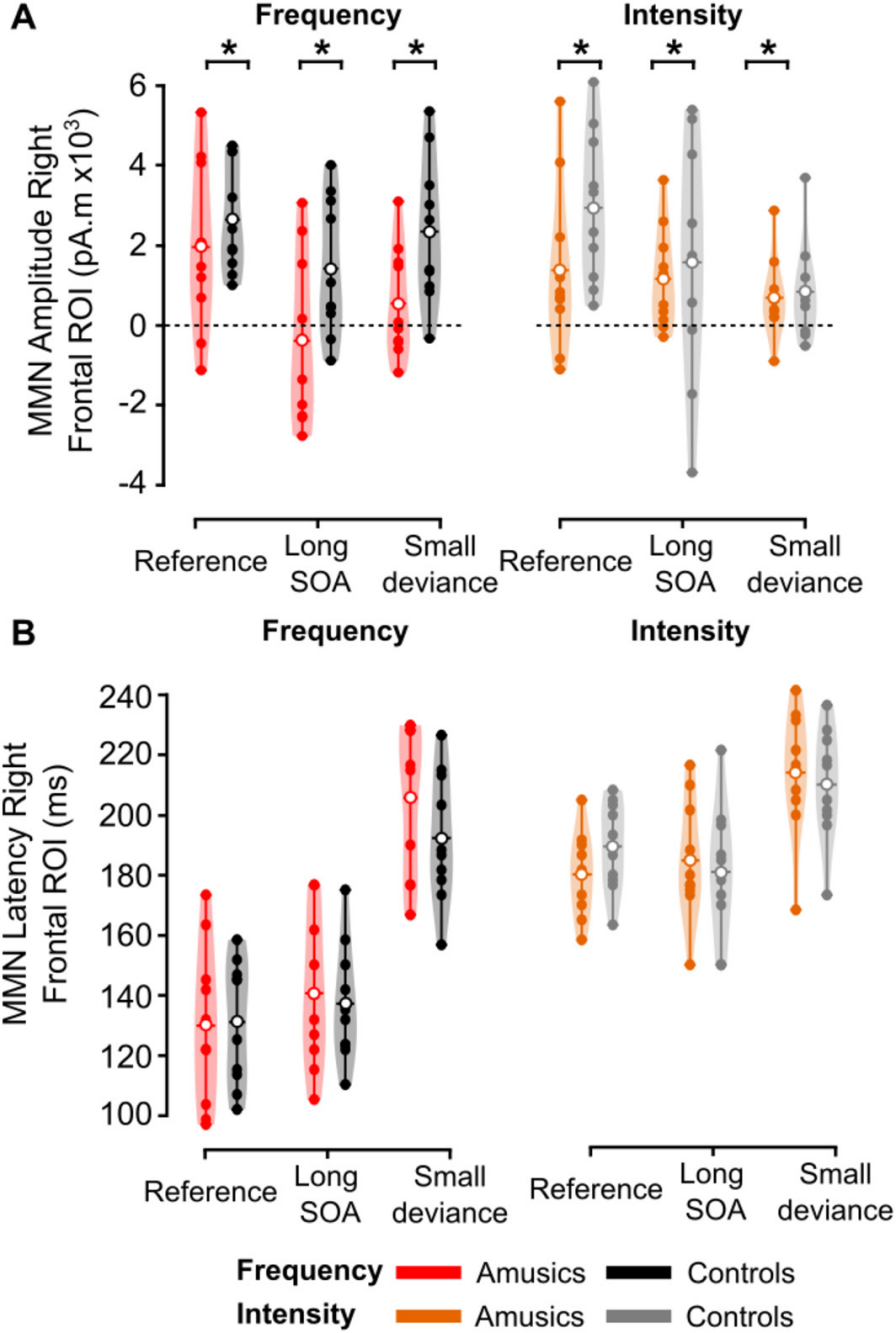
MMNs in the right frontal ROI. (A) Mean source amplitude (in pA·m) and (B) mean peak latency (in ms) in each group. Color code as in Figure 3 represents group and dimension of change: amusics, red and orange violin plots; controls, black and gray violin plots. Filled dots represent individual data, empty dots the mean for each group.

For MMN amplitudes, we found a significant effect of the Group (*F*(1, 36) = 5.59, partial *η* ^2^ = 0.237, *p* = 0.03), with the MMN amplitude reduced in amusic participants compared with control participants. There also was a significant effect of Condition (*F*(2, 36) = 9.16, partial *η* ^2^ = 0.337, *ε* = 0.915, *p* < 0.001): the Long SOA and the Small deviance conditions exhibited a reduced MMN amplitude compared with the Reference condition (*p* < 0.001 and *p* = 0.002 respectively), without a difference between Long SOA and Small deviance conditions (*p* = 0.62). Other effects and interactions were not significant (*p*s > 0.2).

For MMN latencies, the effect of Condition was significant (*F*(2, 36) = 91.53, partial *η* ^2^ = 0.836, *ε* = 0.908, *p* < 0.001), with the Small deviance condition showing significantly increased MMN latency compared with the Reference and to the Long SOA conditions (*p*s < 0.001), without a significant difference between the Reference and the Long SOA conditions (*p* = 0.41). The Dimension effect was also significant (*F*(1, 18) = 157.17, partial *η* ^2^ = 0.897, *p* < 0.001), with increased MMN latency for Intensity deviances compared with Frequency deviances. Dimension interacted with Condition (*F*(2, 36) = 9.34, partial *η* ^2^ = 0.342, *ε* = 0.750, *p* = 0.002), with significantly increased MMN latency for Intensity deviances compared with Frequency deviances for the Reference and Long SOA condition (*p*s < 0.001), but not for the Small Deviance condition (*p* = 0.07). There was no significant effect or interactions involving the Group factor (*p*s > 0.22).

#### 3.2.4 Summary of Between‐Group Differences (Figure 7)

**FIGURE 7:**
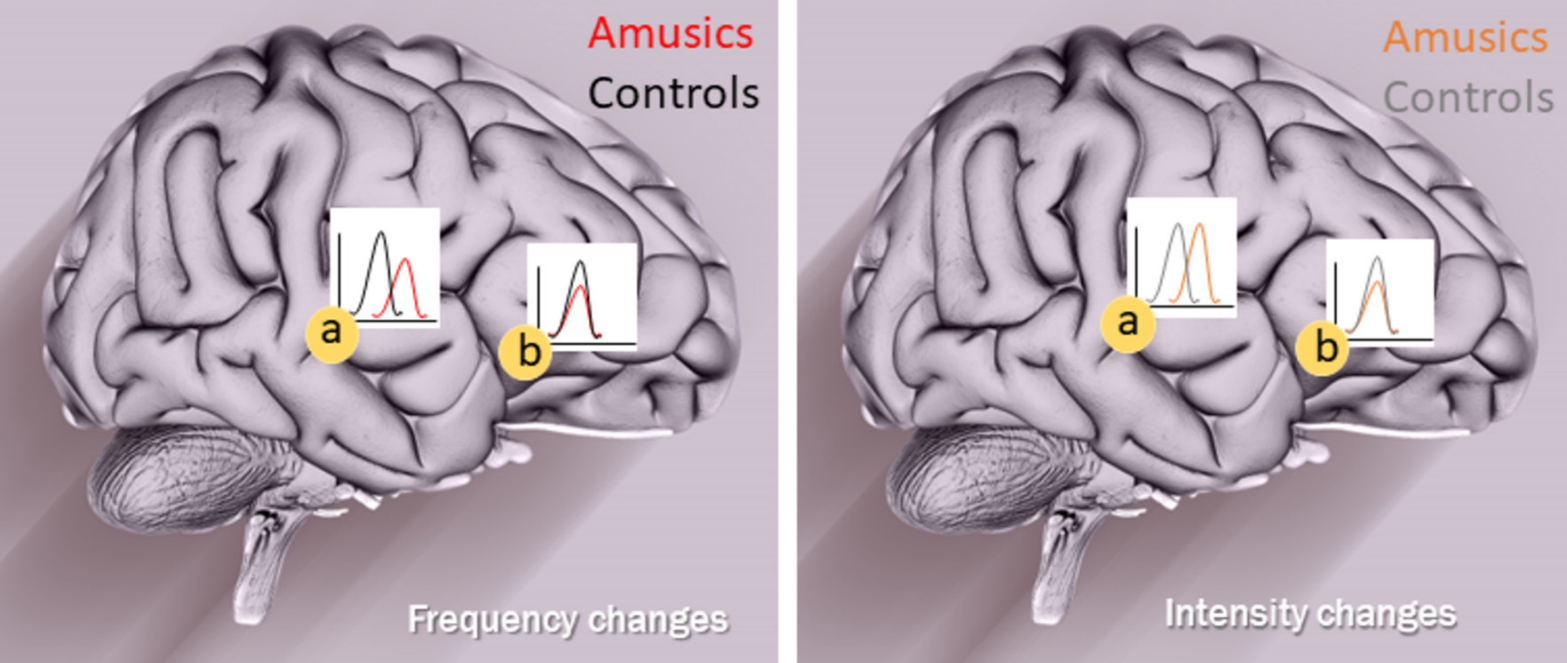
Major effects measured by MEG and contrasting amusics' (in red/orange) and controls' (in black/gray) automatic Frequency change detection (right panel) and Intensity change detection (left panel). MMN amplitude and/or latency differences were observed in the right temporal (a) and the right frontal (b) ROI. The brain was designed by Freepik (https://www.freepik.com/).

The following between‐group differences were thus observed in MMN source data derived from MEG: (1) MMN amplitudes of amusic participants were reduced for Frequency deviants in the right temporal region, in all the conditions, compared with those of controls; (2) also in the right temporal region, MMN latencies of amusic participants were increased compared with those of controls, whatever the condition, for Frequency as well as Intensity; (3) MMN amplitudes of amusic participants were reduced for all deviants in the right frontal region, without between‐group differences in latencies in this ROI; (4) MMN latency tended to increase for amusic participants compared with controls in the left temporal region in the Long SOA conditions, for both Frequency and Intensity deviants.

## 4 Discussion

Using an oddball paradigm in MEG with an active behavioral task (change detection) and a passive listening task involving the same oddball sequences, this study revealed how the detection of changes in a sound sequence is altered in amusia, at both a conscious and preattentive level. As hypothesized, at the behavioral level, congenital amusia was associated with a deficit in active frequency change detection for a small change size as well as for a long SOA. Importantly, this behavioral deficit was associated with alterations in the time course of MMN, which reflects automatic processing, in the right temporal and right frontal regions. Frequency‐MMN amplitude and latency were altered in all tested conditions in participants with amusia, even in the “optimal” (Reference) context. The change size and SOA affected both groups' MMNs. The dominance of a frequency deficit in amusic individuals was clear in the behavioral task, with better abilities at detecting small intensity changes than small frequency changes, while controls presented with the reverse pattern (but amusics always performing worse than controls). Nonetheless, both behavioral data and MMN dynamics revealed that intensity change detection was also impaired in congenital amusia, even though less strongly, with anomalies of MMN latency in the right temporal region and MMN amplitude in the right frontal region. Below we discuss the implication of these findings for our understanding of congenital amusia, of the automatic and explicit mechanisms at stake in change detection, and of the processing of pitch and loudness.

### 4.1 Pitch Change Detection in Congenital Amusia: Automatic Processing Versus Explicit Task Performance

When required to detect pitch changes in an otherwise repetitive sequence, we observed that participants with congenital amusia performed almost at ceiling, as control participants, in an optimal set‐up (large pitch changes of two semitones, SOA of 500 ms). But even with an optimal SOA (i.e., 500 ms), they failed to some extent to detect small pitch changes (of a quarter of a semitone), and increasing the SOA to 1500 ms delayed their response more than that of control participants. These results add to the now rather abundant literature showing pitch processing deficits in congenital amusia, notably in pitch change detection tasks (e.g., Hyde and Peretz 2004; Van Vugt et al. 2025; review in Tillmann et al. 2023), and the fact that these deficits are readily apparent in challenging situations (here decreasing the size of the change or lengthening the SOA). The main question asked in the present study was whether or not these deficits in explicit pitch change detection tasks were accompanied with impaired processing at an automatic, preattentive level, indexed by the MMN. Using MEG recordings, we showed for the first time that it is indeed the case, with reduced amplitude of frequency‐MMNs over all three tested frequency‐deviance conditions in the right temporal cortex, along with increased latency of frequency‐MMNs in the right inferior frontal cortex, compared with control participants' data. These results are in keeping with the known anatomical and functional abnormalities in the right fronto‐temporal network in congenital amusia (review in Peretz 2016; Tillmann et al. 2023), and extend them to automatic pitch change processing. These MEG findings thus highlight impaired automatic extraction of pitch regularities in congenital amusia in a variety of contexts. In optimal contexts like the reference condition, the impoverished memory traces built automatically could be sufficient to achieve a near‐normal behavioral performance in active detection tasks. The involvement of attentional resources could also allow to partially compensate the deficit in automatic stages of processing in such optimal contexts. It is worth noting that the frequency‐MMNs were altered, but were not abolished in congenital amusia. The discrepancy between the current results and previous EEG studies of the MMN in congenital amusia that suggested rather preserved frequency‐MMN (e.g., Moreau et al. 2013; Quiroga‐Martinez et al. 2021) could be due to a higher sensitivity of MEG signals, both thanks to their favorable SNR and the possibility to separate the contribution of distinct generators thanks to source reconstruction (Lecaignard et al. 2021). Indeed, in the present study the EEG data collected simultaneously to the MEG data did not show any significant differences between groups in MMN amplitude or latency (see Supporting Information). This was observed with a standard analysis pipeline for scalp EEG data, notably not including source reconstruction, as in previous EEG studies of the MMN in congenital amusia. The distinct signals from temporal and frontal generators of MMN may be overlapping at the scalp level in EEG, masking subtle differences between groups, whereas MEG source localization effectively separated these sources, allowing to reveal the underlying atypical activity. It must be stressed that a limitation of the current study is sample size (*n* = 10 per group for the EEG and MEG data analyses), calling for future replication studies. The effect sizes for significant Group effects (or interactions involving the group factor) in MEG data analyses as measured by partial *η* ^2^ were between 0.2 and 0.25; and the effect sizes of the non‐significant Group effects in EEG data analyses around a partial *η* ^2^ of 0.15. Effects of such sizes (corresponding to Cohen's *f* > 0.4) would be detected with a power of 80% with larger sample sizes (e.g., 18 participants per group, assuming *f* = 0.4 and *r* = 0.5 for the correlations between three repeated measures, as computed with the G*Power software; Faul et al. 2007). However, with the special population tested, reaching larger sample sizes remains a challenge.

### 4.2 Shared and Distinct Mechanisms for Pitch and Loudness Processing

Using frequency and intensity deviants allowed us to investigate pitch and loudness processing. As expected given previous results on MMN (e.g., Tervaniemi et al. 1999), both types of changes (frequency or intensity) elicited well‐defined MMNs, which became smaller and were delayed when the size of the changes were smaller, or when the SOA within sequences was increased from 500 to 1500 ms (e.g., He et al. 2009). For both dimensions, source reconstruction revealed the involvement of bilateral auditory cortices and right inferior cortices in the generation of the scalp‐recorded signals (as in Lecaignard et al. 2021). However, several features in the result pattern suggested some distinctions between dimensions beyond these anatomical similarities. Namely, reduced MMN amplitudes in the right temporal regions in congenital amusics compared with controls were only observed for frequency‐MMNs, whereas between‐group differences were observed for both frequency and intensity‐MMNs on latencies in the right temporal region and on amplitudes in the right frontal cortex. This result pattern suggests that there could be partly distinct networks for processing pitch and loudness in auditory cortices, whereas frontal areas could be involved more generally in auditory change detection across dimensions. These findings are in keeping with the results of studies that have proposed that frequency‐MMN and intensity‐MMN originate from distinct neural populations in the auditory cortex (e.g., Giard et al. 1995; Rosburg 2003; Lecaignard et al. 2021).

### 4.3 Toward an Integrated View on Congenital Amusia

The present study thus tackled two issues that are still debated for the neurodevelopmental disorder of congenital amusia: firstly, at which processing level does the deficit arise, and secondly, are the anomalies pitch‐specific? The present study reveals that the automatic processing of changes in regular sound sequences is already altered in congenital amusia. This finding is consistent with the atypical early frontal negativity during melodic processing reported in Omigie et al. (2013) with participants' attention being directed toward timbre and not toward melody per se. This might seem somewhat at odds with studies that have revealed rather preserved performance of participants with congenital amusia in implicit/indirect tasks (e.g., Peretz et al. 2009; Tillmann et al. 2014, 2016). However, it should be noted that even if altered, a well‐defined MMN after frequency changes was observed here in participants with congenital amusia, as in previous EEG studies (Moreau et al. 2013; Quiroga‐Martinez et al. 2021, 2022). The quality of automatic sound sequence representation achieved might thus be sufficient to succeed in implicit tasks. Collectively, these findings suggest that atypical auditory sequence processing in amusia is present already in early and automatic stages of processing, with deficient regularity extraction and/or automatic predictive processes. These abnormalities would be carried up and possibly further amplified at later processing stages or depending on the task at hand (see also Zendel et al. 2015). It remains to be analyzed whether, during the course of development, abnormalities at automatic stages of processing impede on the development of higher‐level processing allowing conscious retrieval of pitch information, or the reverse.

Here we further observed abnormalities in processing intensity changes in congenital amusia, which were however more limited that abnormalities in processing frequency changes: (1) at the behavioral level, participants with congenital amusia were impaired compared with controls when detecting small intensity changes, but less so than when detecting small frequency changes; (2) only the frequency‐MMN was reduced in amplitude in right temporal regions in congenital amusia. Several previous studies have highlighted atypical sound processing beyond pitch in congenital amusia (see Tillmann et al. 2023 for a review). Whiteford and Oxenham (2017) reported impaired fine‐grained amplitude modulation detection in amusic compared with control participants. Timbre processing and memory were also found impaired in amusia (Marin et al. 2012; Tillmann et al. 2009), notably the processing of the brightness dimension of timbre (Graves et al. 2019). Temporal and rhythm processing are also impaired in congenital amusia, yet not in all participants with amusia and/or to a smaller extent than pitch processing is impaired (Lagrois and Peretz 2019; Van Vugt et al. 2025). It seems that even if pitch processing anomaly is the hallmark feature of congenital amusia, the deficit can extend to a smaller extent to other auditory dimensions. Based on the present MMN results, we can formulate the hypothesis that the various deficits are associated to a dysfunction in the right prefrontal cortex, where we observed similarly reduced MMN amplitudes for both frequency‐ and intensity‐MMNs in congenital amusia. This also raises the question of when and how in development these anomalies arise, and how dimension‐specific and shared mechanisms for the processing of various auditory features (e.g., pitch, loudness, and timbre) interact during development. Hyde et al. (2007) proposed that thicker cortex observed in auditory and inferior frontal cortex in amusic individuals might be the result of abnormal neural migration during development, altering local connectivity, as proposed also for dyslexia.

To summarize, the present study reveals atypical automatic sound change detection in congenital amusia in a right‐sided fronto‐temporal network, with the largest impairments for frequency changes and smaller impairments for intensity changes. These findings call for future developmental studies to shed light on the interplay between automatic and conscious processing of auditory sequences and the time‐course of typical and atypical development of the fronto‐temporal network.

## Author Contributions

**Yohana Lévêque:** conceptualization, data curation, formal analysis, investigation, methodology, validation, visualization, writing – original draft, writing – review and editing. **Camille Fakche:** data curation, formal analysis, visualization, writing – original draft, writing – review and editing. **Lesly Fornoni:** data curation, investigation, validation, visualization, writing – review and editing. **Françoise Lecaignard:** methodology, software, writing – review and editing. **Sébastien Daligault:** investigation, software, validation, writing – review and editing. **Claude Delpuech:** methodology, software, writing – review and editing. **Julien Jung:** investigation, project administration, writing – review and editing. **Barbara Tillmann:** conceptualization, funding acquisition, methodology, project administration, supervision, writing – review and editing. **Anne Caclin:** conceptualization, data curation, formal analysis, funding acquisition, methodology, project administration, resources, software, supervision, validation, visualization, writing – original draft, writing – review and editing.

## Funding

This work was supported by a grant from “Agence Nationale de la Recherche” (ANR) of the French Ministry of Research ANR‐11‐BSH2‐001‐01 to BT and AC. This work was conducted in the framework of the LabEx CeLyA (“Centre Lyonnais d'Acoustique”, ANR‐10‐LABX‐0060) and of the LabEx Cortex (“Construction, Function and Cognitive Function and Rehabilitation of the Cortex”, ANR‐11‐LABX‐0042) of Université de Lyon, within the program “Investissements d'avenir” (ANR‐11‐IDEX‐0007) operated by the French National Research Agency (ANR). BT was supported by an ANER grant (“MusiC”) of the Region Bourgogne–Franche Comté.

## Conflicts of Interest

The authors declare no conflicts of interest.

## Supporting information


**Figure S1:** MMNs in the left temporal ROI. A. mean source amplitude (in pA·m) and B. mean peak latency (in ms). Color code as in Figure 3 represents group and dimension of change: amusics, red and orange violin plots; controls, black and grey violin plots. Filled dots represent individual data, empty dots the mean for each group.
**Figure S2:** Grand average difference ERPs (deviant‐standard) at Fz for each condition, for each group. Event‐related responses (difference waves) are illustrated in the time window [−100‐400 ms] around sound onset. Negativity is up. Color code as in Figure 3 represents group and dimension of change. Frequency conditions are illustrated in the top panel, intensity conditions in the bottom panel. Line characteristics represent conditions (bold for Reference, plain for Long SOA and bold‐dotted for Small change).
**Figure S3:** MMNs in the EEG data, at Fz. A. mean ERP amplitude (in μV) and B. mean peak latency (in ms) in each group. Color code as in Figure 3 represents group and dimension of change: amusics, red and orange violin plots; controls, black and grey violin plots. Filled dots represent individual data, empty dots the mean for each group.

## Data Availability

The data that support the findings of this study are available from the corresponding author, A.C., upon reasonable request.
