# Supplementary material for: Atypical Change Detection in Sound Sequences: A Behavioral and Magnetoencephalography Study in Congenital Amusia
Source: Eur J Neurosci. 2026 May 4;63:e70529. doi: 10.1111/ejn.70529 (PMC13139517; doi:10.1111/ejn.70529)
Supplement: Supplementary file 1 — Figure S1: MMNs in the left temporal ROI. A. mean source amplitude (in pA·m) and B. mean peak latency (in ms). Color code as in Figure 3 represents group and dimension of change: amusics, red and orange violin plots; controls, black and grey violin plots. Filled dots represent individual data, empty dots the mean for each group. Figure S2: Grand average difference ERPs (deviant‐standard) at Fz for each condition, for each group. Event‐related responses (difference waves) are illustrated in the time window [−100‐400 ms] around sound onset. Negativity is up. Color code as in Figure 3 represents group and dimension of change. Frequency conditions are illustrated in the top panel, intensity conditions in the bottom panel. Line characteristics represent conditions (bold for Reference, plain for Long SOA and bold‐dotted for Small change). Figure S3: MMNs in the EEG data, at Fz. A. mean ERP amplitude (in μV) and B. mean peak latency (in ms) in each group. Color code as in Figure 3 represents group and dimension of change: amusics, red and orange violin plots; controls, black and grey violin plots. Filled dots represent individual data, empty dots the mean for each group. [file EJN-63-0-s001.pdf]

Atypical change detection in sound sequences:
a behavioural and Magnetoencephalography study in congenital amusia

Lévêque Yohana*, Fakche Camille*, Fornoni Lesly, Lecaignard Françoise, Daligault Sébastien, Delpuech Claude, Jung Julien, Tillmann Barbara, Caclin Anne

Supplemental Figure and analysis for the MEG results

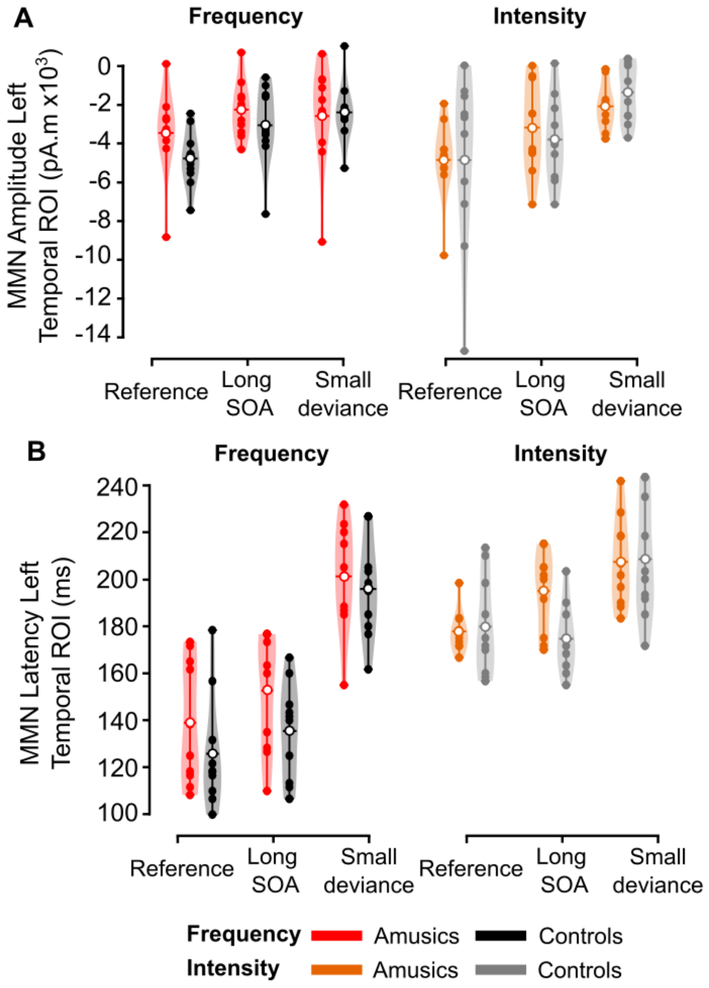

<u>Supplemental Figure S1: MMNs in the left temporal ROI. A. mean source amplitude (in pA.m) and B. mean peak latency (in ms).</u> Color code as in Figure 3 represents group and dimension of change: amusics, red and orange violin plots; controls, black and grey violin plots. Filled dots represent individual data, empty dots the mean for each group.

Statistical analysis of data in the left temporal ROI

The main effect of Condition on MMN amplitude was significant ($F(2,36) = 22.21$, partial $\eta^2 = 0.552$, $\varepsilon = .973$, $p < .001$) and interacted significantly with Dimension ($F(2,36) = 4.19$, partial $\eta^2 = 0.189$, $\varepsilon = 0.892$, $p = .03$). For Frequency-MMNs, there was a difference between the Reference Condition and both the Long SOA ($p = .002$) and the Small deviance ($p < .001$) conditions, but not between the long SOA and Small deviance conditions ($p=.70$), whereas all pairwise comparisons between conditions were significant for Intensity-MMNs (all ps < .004). There was no significant Group effect ($p = .67$) and the Group factor did not interact with other factors (all ps > .19). The Dimension effect was not significant ($p= .60$).

On MMN latencies, the main effect of Condition was significant ($F(2,36) = 107.34$, partial $\eta^2 = 0.856$, $\varepsilon = .859$, $p < .001$). The main effect of Dimension was also significant ($F(1,18) = 102.74$, partial $\eta^2 = 0.851$, $p < .001$), and interacted significantly with Condition ($F(2,36) = 9.36$, partial $\eta^2 = 0.342$, $\varepsilon = .943$, $p < .001$): increased MMN latency for Intensity deviances compared to Frequency deviances were observed in the Reference and Long SOA conditions (ps < .001) but not in the Small deviance condition ($p = .16$). Other effects and interactions were not significant (ps > .05).

Supplemental material: EEG

EEG methods

Recordings were carried out using a 56-electrode EEG system incorporated with the MEG system. The reference electrode was placed on the tip of the nose, and the ground electrode on the shoulder. EEG recordings were made continuously with a sampling rate of 600 Hz and a 0.016 – 150 Hz bandwidth, simultaneously with the MEG, EOG, and ECG recordings.

EEG data preprocessing and analyses

EEG data were pre-processed at the same time as the MEG signals with the same routines, thus exactly the same trials are analysed for EEG and MEG, and data are filtered in the same way, and the same -100 to 0 ms time-window was used as baseline. Emergence t-tests (comparisons to zero of the standard-deviant difference ERPs) were run at each sample at Fz across all participants separately for each condition. The time windows of significant MMN emergence were used for ERP analyses (Frequency Reference: 103 - 178 ms; Frequency Long SOA: 95 - 190 ms; Frequence Small deviance: 166 - 216 ms; Intensity Reference: 138 - 216 ms; Intensity Long SOA: 158 - 233 ms; Intensity Small deviance: 206 - 226 ms). For each participant, each condition, and each dimension, we extracted MMN mean amplitude and latency at Fz, in the time window of interest, as for the analyses of the MEG source data (the MMN has negative polarity at Fz, thus the latency of the minimum was retrieved). Three-way repeated-measure ANOVAs were performed on MMN mean amplitude and MMN latency measured at Fz (see Supplemental Figure 2 for the time course of ERPs at Fz), with the between-participant factor Group (Amusic/Control), and within-participant factors Dimension (Frequency/Intensity) and Condition (Reference/Long SOA/Small deviance).

Results

MMN amplitudes and latencies at Fz are depicted in Supplemental Figure 3. For MMN amplitudes, the effect of Group had a large effect size but did not reach statistical significance ($F(1,18) = 3.50$, partial $\eta^2 = 0.162$, $p=.078$), with amusic participants showing reduced amplitude compared to control participants. The interaction between Dimension, Condition, and Group had a moderate effect size and did not reach statistical significance ($F(2,36) = 2.65$,

partial $\eta^2 = 0.129$, $\varepsilon = 0.970$, $p = .09$). The effect of Dimension had a large effect size but did not reach significance ($F(1,18) = 3.04$, partial $\eta^2 = 0.145$, $p=.10$), with larger MMN amplitudes in Intensity compared to Frequency conditions. There was no other significant effect or interaction (ps>.31).

For MMN latencies, the main effect of Dimension ($F(1,18) = 138.55$, partial $\eta^2 = 0.885$, $p< .001$) was significant, MMN latencies were larger for Intensity than for Frequency deviances. The main effect of Condition ($F(2,36) = 47.59$, partial $\eta^2 = 0.726$, $\varepsilon = 0.841$, $p < .001$) was significant: MMN latencies were larger in the Small change conditions than in the Reference and Long SOA conditions (ps<0.001), without a significant difference between the Reference and Long SOA conditions (p=.39). The interaction between Dimension and Condition ($F(2,36) = 6.82$, partial $\eta^2 = 0.275$, $\varepsilon = 0.975$, $p = .003$) was significant, with differences in MMN latencies between the Reference and Long SOA conditions being significant for the Intensity MMN (p=.008), but not for the Frequency MMN (p=.21), other pairwise comparisons between conditions are significant for both dimensions (ps<.001). The effect of Group had a large effect size but did not reach statistical significance ($F(1,36) = 3.38$, partial $\eta^2 = 0.158$, $p = .08$), with longer MMN latencies in amusics compared to controls. There was no other significant effect or interaction (ps>.33).

Overall, while the EEG result pattern somewhat mirrors the MEG source results, showing smaller and delayed MMNs in amusics compared to controls, effects and interactions involving the Group factor in EEG scalp data did not reach statistical significance (all p-values > 0.07). We reported those here in addition to the significant effects as they provide converging evidence with the MEG data, further supporting its increased sensitivity.

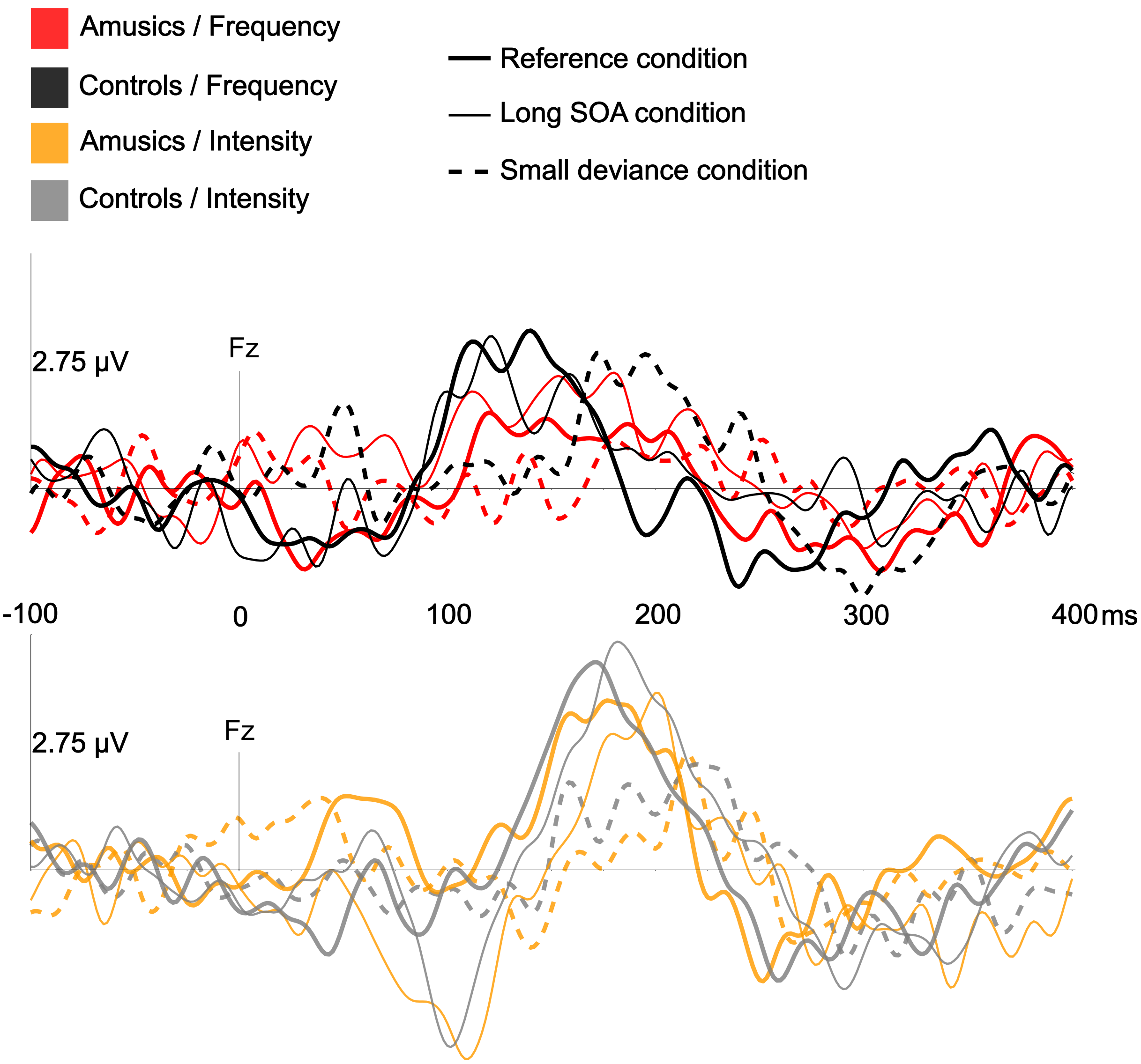

<u>Supplemental Figure S2: Grand average difference ERPs (deviant-standard) at Fz for each condition, for each group</u>. Event-related responses (difference waves) are illustrated in the time window [-100-400ms] around sound onset. Negativity is up. Color code as in Figure 3 represents group and dimension of change. Frequency conditions are illustrated in the top panel, intensity conditions in the bottom panel. Line characteristics represent conditions (bold for Reference, plain for Long SOA and bold-dotted for Small change).

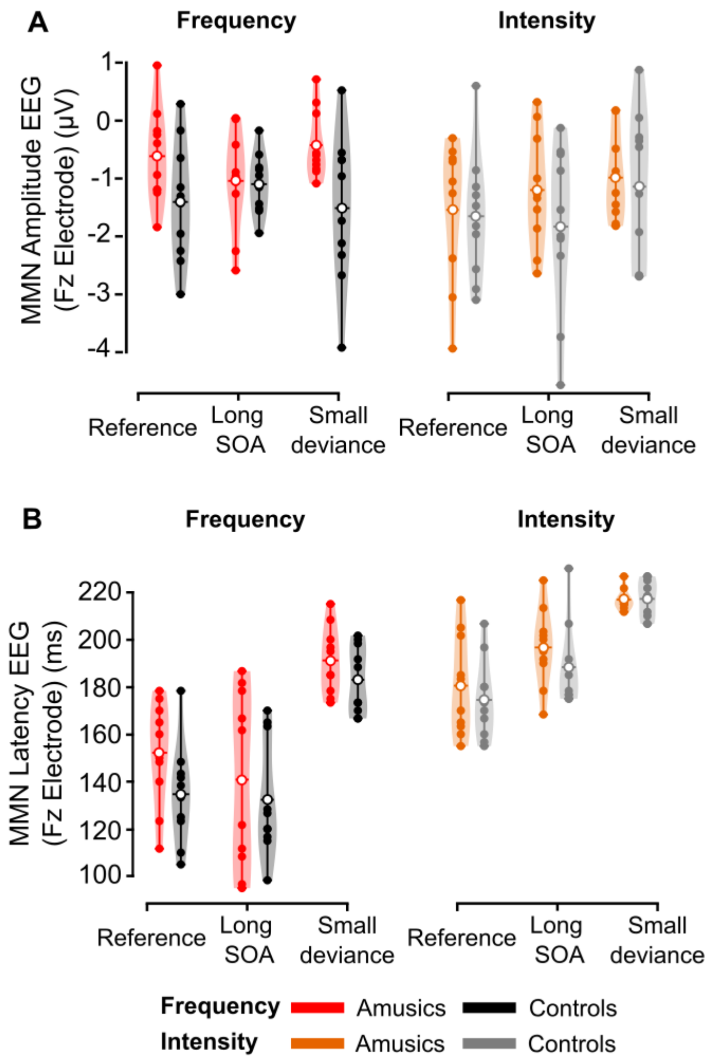

Supplementary Figure S3: MMNs in the EEG data, at Fz. A. mean ERP amplitude (in µV) and B. mean peak latency (in ms) in each group. *Color code as in Figure 3 represents group and dimension of change: amusics, red and orange violin plots; controls, black and grey violin plots. Filled dots represent individual data, empty dots the mean for each group.*
